# The Physiology and Pathology of the Mouth.—The Glands and Their Secretions

**Published:** 1860-02

**Authors:** 


### DENTAL REGISTER OF THE WEST.

VOL. XIII.] FEBRUARY, 1860. [No. 8.

### Original Essays and Communications.

### THE PHYSIOLOGY AND PATHOLOGY OF THE MOUTH.—THE GLANDS AND THEIR SECRETIONS.

THE *glands* within and about the mouth are very numerous, and the amount of fluid which they pour out into that cavity is very great—far more than passes into the blood from food or drink, rapid as is endosmotic absorption in this part of the system ; and hence a considerable quantity passes down the throat, both with our food and at other times. The amount of fluid poured into the mouth by the glands, more than is re-absorbed while in the mouth, has been estimated by Bidder and Schmidt, of Derpt, as detailed in their great work *on the Digestive Juices*, at from three to four pounds, avoirdupois, of saliva in the twenty-four hours.

The quantity of saliva poured out varies greatly in amount at different times and under different circumstances. Not only is its quantity changed in accordance with the nature of the food eaten, but also in accordance with the mental impressions. But very little is secreted during sleep, but the thought of food will frequently fill the mouth to overflowing almost instantly. Dentists are well aware that if they can get the attention of their patients, they can sometimes perform operations with but little interruption from the saliva, and yet when once it commences to flow, it will do so abundantly for a considerable time.

As has been intimated, the nature of the food also has considerable influence upon the quantity of saliva secreted; hard, dry foods leading to a much greater flow than is caused by those which are soft and moist. There are several articles which are used as condiments, or seasoning, with food, which also appear to be direct stimulants to the glands that secrete saliva and water into the mouth. It is well known that black, but more especially red pepper, produces this effect in a marked degree. So also, probably, does salt, and it also causes free exosmosis of the fluid portion of the blood out through the buccal mucous membrane. The movement of the jaws, likewise, as in speaking or chewing, increases the amount of this secretion, and frequently hunters and others who are suffering some inconvenience from thirst, chew a piece of stick or a small coin, in order that its mechanical impressions, and the movement of the jaws and the muscles it causes, should lead to an increase of the moisture within the mouth. The usual manipulations of the dentist have been observed to increase the flow of these fluids at least to ten-fold their ordinary amount, but the excitement continues but a short time.

The *salivan* glands are not the only glands whose secretions are poured into the buccal cavity. The entire mucous lining of the mouth may be considered as a secreting glandular surface, for *all* secreting glands are but, as it were, an enfoldment of a secreting surface crumpled up into a small compass, as a sheet of paper may be enfolded and crumpled up in the hand so as to be made to assume a globular form.

In the remarks that have been made respecting the *Epethelium*, it has been considered simply as a covering to the mucous membrane. So also in regard to *osmosis*, the blood-vessels and their contents were mainly considered, and with reference, principally, to the osmotic flow of fluids into the blood-vessels from that taken into the mouth as food or drink. It is proper now to consider the mucous membrane with reference to its peculiar secretion.

The word *Mucus* conveys to the reader, generally, but a vague idea. It is a vague term. We are led, usually, to suppose that a technical term of Latin origin has a distinct and definite meaning, but no very definite meaning is usually attached to this word. Perhaps the word *slime* would have been preferable, as that would imply only the one quality of a glassy, semi-jelly-like fluid, easily drawn into strings or threads, powerfully coherent, and very tenacious in its attachment to any surface it is in contact with.

The chemist can not tell us whether pure mucus is acid or otherwise—or whether the soda often found with it is a necessary part of it, and it is therefore alkaline. The microscopist describes some variously shaped bodies he observes through his lenses, and calls them "mucus globules," but these bodies occur in greatly varying proportionate quantities, sometimes one form or size, constituting the greater proportion of the solid matter within the fluid we call mucus, and sometimes another form or size being in similar excess, and yet apparently having but a little if any influence on the nature of the fluid.

It is quite evident, however, that some of these bodies observed through the microscope, are detached epithelial scales, only differing from each other in accordance to the usual variation in form to be found in epithelial scales from different portions of the mucous membrane. Other of the so-called "mucus globules" appear to be simply epithelial scales not fully perfected before they have become detached—youthful epithelial scales—such as are always to be seen in the deeper parts of the mucous membrane, being nearly round balls or beads of jelly, with one jelly bead for a center or nucleus, which is quite large and with distinct outlines, and that surrounded with several other similar bodies. These two forms of bodies—the apparently dead old epithelial scales, and the youthful ones—are so constantly present in mucus, and in such large quantities as to be supposed by some to be *the* mucus; and the water present is considered simply as water in which the mucus is suspended, or which the mucus has absorbed.

The slightest irritation, and of a strictly healthy character, increases vastly, not only the amount of water in the mouth, but also of the salts that water holds in solution, as well of the other matters in the water. Many have supposed that the epithelial scales were the mucus, and that they absorbed the water as trajacunth does, and thus became the glassy, jelly-like semi-solid known by that name; while others supposed the fluid was mucus, before the epithelial scales were combined with it, and that they should be considered as accidental, or at least foreign bodies, and not a part of the mucus, *per se.* Dr. Tilamis, Prof. Rokistanki, Virchow, and other eminent physiologists, have inclined to the opinion that the peculiar characteristics of mucus, or that which makes it mucus, is the old and the young epithelial scales and globules become detached and filled to saturation with water.

But from whatever source it comes, mucus is a glassy, half-jelly like fluid, transparent, easily drawn out, tough, adherent; that it is capable of absorbing a vast amount of water which swells it up—but is not capable of being dissolved in it, for mucus is proved to be entirely insoluble and only sparingly diffusible in water. Its toughness and stringiness is in proportion to the amount of "mucus globules" in it if not produced by them.

The mucus of the mouth, as well as in other parts of the system, is formed by both the free mucous surface, and also by special mucous glands. When formed by special glands, it is usually mixed with *solvents* designed to dissolve foods, and which are to be considered hereafter. The mucus thrown off from the free surface, does not appear to be mixed with solvents.

As mucus is but slightly diffusible in water, it is not washed away in the fluids of the mouth; but is spread over the surface from which it is secreted, and adheres to it with considerable tenacity, and there acts on many substances, chemically, in a way that fits them to be readily absorbed through the membrane, while its tenacity of adhesion, to the membrane, its slipperiness and elasticity seem to make it a most admirable shielder to the epithelium against the mechanical or chemical injuries that delicate pavement is liable to.

As the mucus absorbs a large amount of water, it serves to keep the membrane *moist*, which is an essential pre-requisite to osmosis, in both directions. Without being moist, the membrane would lose nearly all its functional uses—and mucus is the great cause of its constant moisture.

*The Saliva*, as a fluid, almost constantly present in the mouth, has always attracted a considerable share of the attention of the physiologist, but until quite recently no one appeared, even approximately, to determine its quantity, and were not quite decided as to its uses. But Drs. Bidder and Schmidt have carefully guarded against errors in their numerous experiments and have concluded that about three pounds is the amount secreted in man in twenty-four hours.

The amount of saliva excreted in a given time varies with the state of the mind, and the nature of the food taken. It is also greatly affected by the use of stimulants, which act to increase the flow for a considerable period of time after they are withdrawn. The *quality* of the saliva seems to be but little, if any affected, even when it is greatly changed in amount. But the different salivary glands secrete a very different amount of *water*, or saliva of differing degrees of dilution. Bernard, Bidder and Schmidt, and others have always found the fluid secreted by the parotid glands so very *watery*, or so little of other matter besides water, that they have come to the conclusion that they are truly *glandes aquipares*, and not true salivary glands. Mr. Jacrebowitsch found only 4-7 parts of solid matter to 1000 parts of the excretion of the parotid glands; but the fluid secreted from all the glands and the mucous surface of the mouth contained 10-37 parts of solid matter to the 1000 parts of fluids.

As there has been experienced great diffculty in determining the amount of saliva secreted, so also has there been great difficulty in determining its chemical character.

Sulpho-cyanide of potassium has been long known to exist in the saliva, and it is found in abundance in proportion to the vigor and healthful activity of the individual secreting it. Yet its use remains to this day utterly undetermined. Dr. Kletzinsky suggests that it is to prevent the mouth from becoming mouldy! It does not, as far as can be ascertained, promote digestion, or any other change in the food.

In nearly all works on physiology there are tables of chemical analysis, showing the different chemical bodies that enter into the composition of the saliva, but however valuable they may be in determining the chemical and electrical actions and reactions which will follow the introduction of the various metals and alloys into the mouth, by the dentist, they do not seem in the least degree important in determining the physiological action and uses of saliva, for those physiological actions do not appear to be modified in the slightest degree by the amount, or presence, or absence of any one ingredient. Hence, physiologically considered, the saliva, may be spoken of, simply as a *fluid*, as milk, may be spoken of as a fluid.

In regard to the *Dental Chemistry of the Mouth*, nothing need now be said, as that matter has been ably and fully presented through the colums of the *Register*, by Prof. Watt.

The saliva possesses the power of forming sugar out of amylaceous, or starchy food. This is not a newly discovered power of saliva, but the recent experiments in the Derpt laboratory have fully established the fact, that had come to be doubted by some. This conversion of starch into sugar appears to commence *instantaneously* on the union of saliva and a decoction of starch, whether the mixture takes place in the mouth or in a glass vessel, and hence is a purely chemical change. The pancreatic and intestinal juices, possess this power of conversion also, in common with the saliva; unboiled or *raw* starch is not thus changed.

Neither the secretions of the parotid glands, the sub-maxillary and sublinguial glands, or the buccal mucous membrane, when taken separately possess this power of converting starch into sugur *and only an admixture of them*, will produce a change—or at least an admixture of the buccal mucus with fluid from the sub-maxillary glands. The parotid fluid seems to be simply water. When the saliva is considerably acid, as from disease, the starch is not changed into sugar as quickly, and yet it is changed after a time.

The present state of our knowledge in regard to changes produced by the saliva on the food, indicates that boiled or cooked starchy food, is in part at least changed by the saliva into sugar; that a part of this sugar may be at once absorbed from the mouth into the system, and a larger portion passes into the stomach, there to be changed into lactic acid. The other part of the starch, not changed into sugar in the mouth, may be exchanged by contact with the pancreatic and intestinal juices, for they possess the same power as the saliva.

The saliva is not known to produce any other metamorphosis than that of changing starch in sugar; but it does *retard* the digestion of meats, and animal food or albumen, and hence such foods are not usually retained long in the mouth, or much mixed with saliva. Quite young infants do not secrete any saliva.

As saliva has for its source many parts of the mouth, an injury to any one part of it is not likely to seriously modify its amount. These glands also have great power of accommodating themselves to quite considerable changes of circumstances, and hence it is that the disgusting habit of spitting indulged in by some, does not appear to produce serious suffering to those who *do not* expectorate as gentlemen, however much others may be made to suffer by it. Others do suffer from a loss of appetite, and a feeling of distress at the epigastrium. Those who use tobacco, doubtless suffer less from the loss of saliva than they would from swallowing the poisonous juice of the weed.

When the tongue, lips, and mucous membrane become dry and parched, and the teeth covered with a thick sordes, as in some forms of fever, starchy food is highly improper, for then there is not enough starch secreted to change the saliva into sugar, and it therefore can not be changed into a soluble substance to be absorbed into the system. Then beef-tea, broths, and soups, will be beneficial, and almost always agreeable to the palate.

